# Low prevalence of highly sulfadoxine‐resistant dihydropteroate synthase alleles in *Plasmodium falciparum* isolates in Benin

**DOI:** 10.1186/s12936-021-03605-5

**Published:** 2021-02-05

**Authors:** Samaly Souza Svigel, Adicath Adeothy, Augustin Kpemasse, Ernest Houngbo, Antoine Sianou, Ramani Saliou, Monica E. Patton, Fortune Dagnon, Eric S. Halsey, Alexis Tchevoede, Venkatachalam Udhayakumar, Naomi W. Lucchi

**Affiliations:** 1 Malaria Branch, Division of Parasitic Diseases and Malaria, Centers for Disease Control and Prevention, Atlanta, GA USA; 2 National Malaria Control Programme, Ministry of Health, Cotonou, Benin; 3 Accelerating the Reduction of Malaria Morbidity and Mortality Project (ARM3), Medical Care Development International, Cotonou, Benin; 4 U.S. President’s Malaria Initiative, USAID, Cotonou, Benin; 5 U.S. President’s Malaria Initiative, GA Atlanta, USA; 6 Present Address: John Snow, Inc. (JSI) , MA Boston, USA

**Keywords:** Drug resistance, Sulfadoxine Pyrimethamine, *Pfdhfr*, *Pfdhps*, Intermittent Preventive Treatment in Pregnant, Seasonal Malaria Chemoprevention, Pregnant women, Malaria

## Abstract

**Background:**

In 2004, in response to high levels of treatment failure associated with sulfadoxine-pyrimethamine (SP) resistance, Benin changed its first-line malaria treatment from SP to artemisinin-based combination therapy for treatment of uncomplicated *Plasmodium falciparum* malaria. Resistance to SP is conferred by accumulation of single nucleotide polymorphisms (SNPs) in *P. falciparum* genes involved in folate metabolism, *dihydrofolate reductase (Pfdhfr)* and *dihydropteroate synthase (Pfdhps)*, targeted by pyrimethamine and sulfadoxine, respectively. Because SP is still used for intermittent preventive treatment in pregnant women (IPTp) and seasonal malaria chemoprevention (SMCP) in Benin, the prevalence of *Pfdhfr* and *Pfdhps* SNPs in *P. falciparum* isolates collected in 2017 were investigated.

**Methods:**

This study was carried out in two sites where the transmission of *P. falciparum* malaria is hyper-endemic: Klouékanmey and Djougou. Blood samples were collected from 178 febrile children 6–59 months old with confirmed uncomplicated *P. falciparum* malaria and were genotyped for SNPs associated with SP resistance.

**Results:**

The *Pfdhfr* triple mutant **IRN** (N51**I**, C59**R**, and S108**N**) was the most prevalent (84.6%) haplotype and was commonly found with the *Pfdhps* single mutant A437**G** (50.5%) or with the *Pfdhps* double mutant S436**A** and A437**G** (33.7%). The quintuple mutant, *Pfdhfr* **IRN**/*Pfdhps* **GE** (A437**G** and K540**E**), was rarely observed (0.8%). The A581**G** and A613**S** mutant alleles were found in 2.6 and 3.9% of isolates, respectively. Six isolates (3.9%) were shown to harbour a mutation at codon I431**V**, recently identified in West African parasites.

**Conclusions:**

This study showed that *Pfdhfr* triple **IRN** mutants are near fixation in this population and that the highly sulfadoxine-resistant *Pfdhps* alleles are not widespread in Benin. These data support the continued use of SP for chemoprevention in these study sites, which should be complemented by periodic nationwide molecular surveillance to detect emergence of resistant genotypes.

## Background

Malaria is a major health problem in Benin and is the leading cause of mortality among children under 5 years of age and morbidity among adults. In 2019, the World Health Organization (WHO) reported an estimated four million malaria cases and over 7000 deaths in the country [1]. In 2004, Benin joined many other countries in Africa in changing their recommended first-line treatment of uncomplicated malaria to artemisinin-based combination therapy (ACT) [2] due to reported high treatment failure rates in children treated with sulfadoxine-pyrimethamine (SP) for uncomplicated malaria [3–5]. However, the WHO recommends the continued use of SP for intermittent preventive treatment in pregnant women (IPTp) [6], as well as for seasonal malaria chemoprevention (SMC), used in combination with amodiaquine (SP-AQ) for the latter indication, in countries with highly seasonal malaria transmission such as the Sahel region of sub-Saharan Africa [7].

Resistance to SP is conferred by accumulation of single nucleotide polymorphisms (SNPs) in two genes that code for enzymes involved in *Plasmodium falciparum* folate metabolism: *P. falciparum* dihydrofolate reductase (*Pfdhfr*) and *P. falciparum* dihydropteroate synthase (*Pfdhps*), which are targeted by pyrimethamine and sulfadoxine, respectively. At least five mutations in *Pfdhfr* confer resistance to pyrimethamine: C50**R**, N51**I**, C59**R**, S108**N**, and I164**L** (amino acid substitutions in bold face). Similarly, at least five SNPs in *Pfdhps* are involved in resistance to sulfadoxine: S436**A/F**, A437**G**, K540**E**, A581**G**, and A613**S/T** [8–12].

The combination of *Pfdhfr* triple mutant **IRN** with the *Pfdhps* double mutant **GE** results in a quintuple mutant, which has been shown to lead to clinical treatment failure of SP [13–15]. In general, these quintuple mutants are commonly found throughout East Africa, but rarely in West and Central Africa [16–18]. In contrast, the *Pfdhfr* triple mutant **IRN** with the *Pfdhps* A437**G** is often found in West Africa and is also associated with treatment failure, but to a lesser degree than the **IRN** plus **GE** quintuple mutants [16–21]. Studies have demonstrated that the efficacy of SP for IPTp is still acceptable even when a high prevalence of SP resistance markers exists, including the **IRN** plus **GE** quintuple mutants and the **IRN** plus **G** quadruple mutants [6, 22–25], justifying the continued use of SP for IPTp and SMC. However, the occurrence of additional *Pfdhps* mutations at codons A581**G** [19, 26–28] and A613**S/T** [27] or *Pfdhfr* I164**L** [29, 30] to the quintuple **IRN** plus **GE** mutant genotype was shown to lead to declines in SP’s IPTp efficacy [28] and protection in infants [31].

Studies of molecular markers of SP resistance in Benin carried out between 2003 and 2012 in the north, south [32–34], and the coast [4, 35, 36] showed that the majority (> 90%) of parasites carry **IRN** *Pfdhfr* mutations. The most common mutation in *Pfdhps* was A437**G** (71.4%) [34]. A low prevalence of K540**E** (8.3%) was found [36] and no mutation was found at codon S436**A** in a study conducted between 2008 and 2010 [37]. However, recent data on molecular markers associated with SP resistance is lacking. This study investigated the prevalence of SNPs in *Pfdhfr* and *Pfdhps* in *P. falciparum* isolates collected from Benin in 2017.

## Methods

### Study population and sample collection

**Fig. 1:**
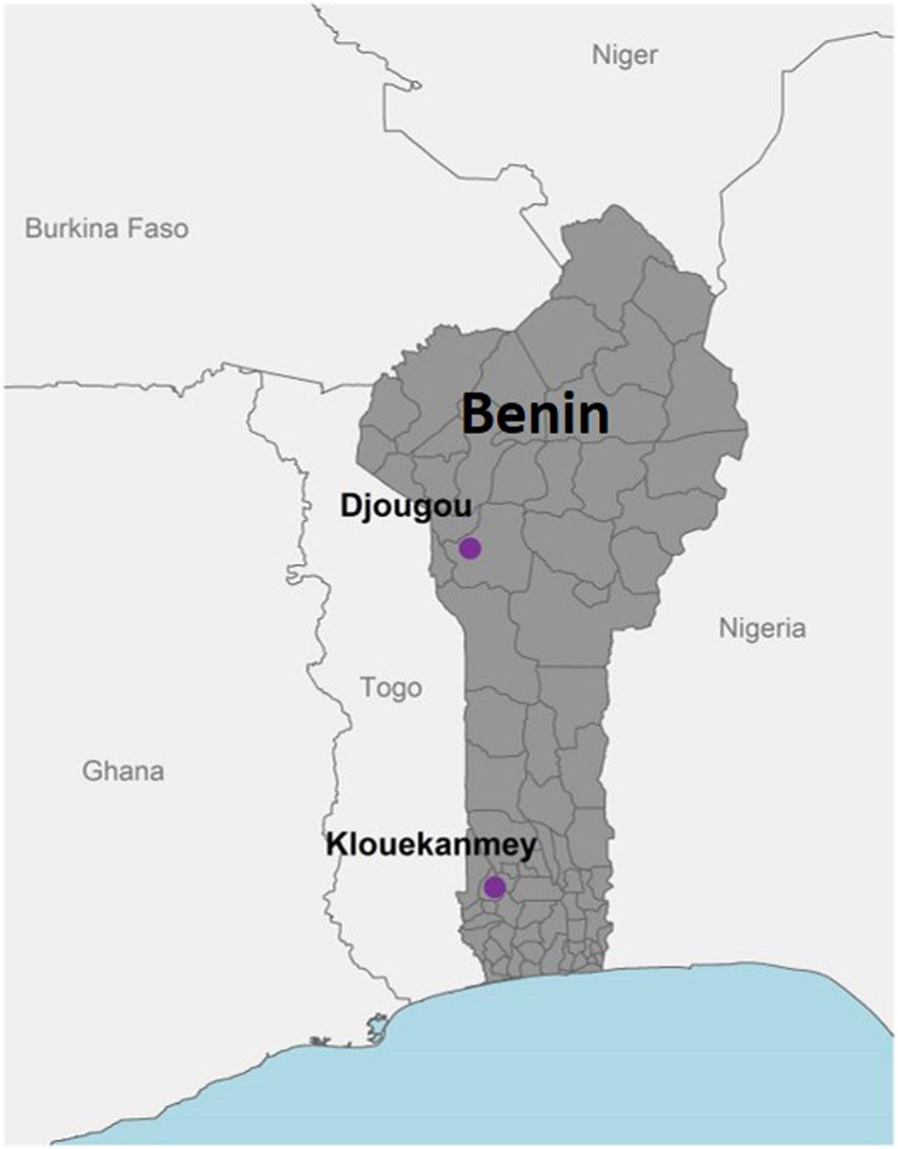
Location of study sites used for the therapeutic efficacy study, Benin, 2017. Benin map (shaded in gray) indicating the location of the two sentinel sites, Klouékanmey and Djougou (purple dots), in which samples were collected and used to determine the prevalence of sulfadoxine-pyrimethamine resistance markers in *Plasmodium falciparum* isolates

The samples utilized in this study were obtained from a therapeutic efficacy study (TES) of artemether-lumefantrine conducted by the National Malaria Control Programme (NMCP) in Benin in 2017 (results unpublished). The study was carried out in two NMCP sentinel sites, Klouékanmey and Djougou, where the transmission of *P. falciparum* malaria is hyper-endemic (Fig. 1). Criteria for inclusion included children 6–59 months old with monoinfection of *P. falciparum*, measured by microscopy, a parasite density between 2000 and 200,000 parasites/µl, axillary temperature of 37.5 °C or higher, and ability to take oral medication. Children with signs of severe illness and malnutrition were excluded. Enrolled patients were treated with a supervised 3-day course of artemether-lumefantrine and monitored for 28 days with weekly scheduled visits on days 7, 14, 21, and 28. Patients were also asked to return to the clinic if they became ill any other day during the follow-up period (unscheduled visits). Dried blood spots (DBS) were collected on Whatman grade 3 filter paper (GE Healthcare Life Sciences, Marlborough, USA) from enrolled patients on the day of enrollment (pre-treatment) and on the scheduled and unscheduled visits. Only samples from the day of enrollment were utilized in this study.

### **Sample processing and*Pfdhfr*and*Pfdhps*molecular analysis**

Genomic DNA was isolated from the DBS using the QIAamp® blood mini kit (Qiagen Inc., CA, USA) per the manufacturer’s recommendations. The *Pfdhfr* and *Pfdhps* gene fragments were amplified by polymerase chain reaction (PCR) using previously published primers [38]. SNPs at *Pfdhfr* codons 50, 51, 59, 108, and 164 and *Pfdhps* codons 431, 436, 437, 540, 581, and 613 were investigated using Sanger sequencing as previously described [38]. The PCR products were precipitated in 70% ethanol to clean up dye terminators, rehydrated in 10 µl Hi-Di Formamide™, and sequenced using the Applied Biosystems 3130xl sequencer (Life Technologies, Grand Island, NY). Sequences were analysed using Geneious software (Biomatters, San Francisco, CA, USA). The 3D7 *P. falciparum Pfdhps* (Gene ID: 2,655,294) and *Pfdhfr* (Gene ID: 9,221,804) were used as reference sequences in the analysis.

## Data management and analysis

Data were entered into a Microsoft Excel database and descriptive statistics such as percentage, mean, and range were reported as appropriate. The prevalence of different alleles and haplotypes in the *Pfdhfr* and *Pfdhps* genes were reported per site. The prevalence of the different alleles was reported as wild type (having only the wild type allele), mutant (having only the mutant allele), or mixed infection (having both wild type and mutant alleles).

## Results

A total of 178 pre-treatment samples (85 from Klouékanmey and 93 from Djougou) were evaluated for molecular markers of resistance in the *Pfdhfr* and *Pfdhps* genes. The mean age of the patients was 33 (SD = 14) months and 62.1% were male. The geometric mean *P. falciparum* parasite density was 24,154 (95% CI 16,600–31,700); range: 2,081–200,000) parasites/µl.

### **Prevalence of*Pfdhfr and Pfdhps*alleles (SNPs)**

A total of 169 (94.9%) specimens were successfully sequenced for the *Pfdhfr* gene. Twelve samples (7.1%) had a mixed infection. No mutations were found at codons 50 or 164. Overall, a high prevalence of mutations was observed in codons N51**I**, (86.4%; 146), C59**R**, (89.9%; 152), and S108**N**, **(**94.7%; 160); an additional 4.1, 6.5, and 2.4% of samples, respectively, contained both mutant and wild-type alleles as part of a mixed infection, Table 1. For the *Pfdhps* gene, 153 (86.0%) samples were successfully sequenced and 21 of these (13.7%) had a mixed infection. Overall, a majority of the samples had the A437**G** mutation (94.8%; 145) followed by the S436**A** mutation (35.3%; 54), Table 1. All samples from Klouékanmey (100%; 68) had the A437**G** mutation compared to 95.3% (81) in Djougou, of which four (4.7%) were mixed alleles. The A581**G** and A613**S** alleles were observed in 2.6 and 3.9% of isolates, respectively, with an additional sample from Djougou having a 613A/**S** mixed infection. Six isolates (3.9%) were shown to harbour a mutation at codon I431**V**, three from Klouékanmey and three from Djougou, of which two were mixed with wildtype parasites, Table 1.

**Table 1:** Summary of alleles observed in *Pfdhfr* and *Pfdhps* genes

*Pfdhfr*	Klouékanmey n = 79 (%)	Djougou n = 90 (%)	Overall n = 169 (%)
C50	79 (100)	90 (100)	169 (100)
50**R**	0	0	0
50 C/**R**	0	0	0
N51	3 (3.8)	13 (14.4)	16 (9.5)
51**I**	72 (91.1)	74 (82.3)	146 (86.4)
5**1** N**/I**	4 (5.1)	3 (3.3)	7 (4.1)
C59	1 (1.3)	5 (5.6)	6 (3.6)
59**R**	72 (91.1)	80 (88.8)	152 (89.9)
59 C/**R**	6 (7.6)	5 (5.6)	11 (6.5)
S108	0	5 (5.6)	5 (3.0)
108** N**	78 (98.7)	82 (91.1)	160 (94.7)
108S/**N**	1 (1.3)	3 (3.3)	4 (2.4)
I164	79 (100)	90 (100)	169 (100)
164**L**	0	0	0
164I/**L**	0	0	0

*Pfdhps*	Klouékanmey n = 68 (%)	Djougou n = 85 (%)	Overall n = 153 (%)
I431	65 (95.6)	82 (96.4)	147 (96.1)
431**V**	3 (4.4)	1 (1.2)	4 (2.6)
431I/**V**	0	2 (2.4)	2 (1.3)
S436	41 (60.2)	40 (47.1)	81 (52.9)
436**A**	22 (32.4)	32 (37.6)	54 (35.3)
436S/**A**	5 (7.4)	13 (15.3)	18 (11.8)
A437	0	4 (4.7)	4 (2.6)
437**G**	68 (100)	77 (90.6)	145 (94.8)
437A/**G**	0	4 (4.7)	4 (2.6)
K540	66 (97.0)	83 (97.6)	149 (97.4)
540**E**	1 (1.5)	1 (1.2)	2 (1.3)
540K/**E**	1 (1.5)	1 (1.2)	2 (1.3)
A581	65 (95.6)	84 (98.8)	149 (97.4)
581**G**	3 (4.4)	1 (1.2)	4 (2.6)
581A/**G**	0	0	0
A613	65 (95.6)	81 (95.3)	146 (95.4)
613**S**	3 (4.4)	3 (3.5)	6 (3.9)
613A/**S**	0	1 (1.2)	1 (0.7)

Bold letters denote mutant alleles

## Observed haplotypes per gene

Table 2 summarizes the observed haplotypes for the *Pfdhfr* and *Pfdhps* genes. Haplotypes were constructed using codons C50**R**, N51**I**, C59**R**, S108**N**, and I164**L** in the *Pfdhfr* gene and I431**V**, S436**A**, A437**G**, K540**E**, A581**G**, and A613**S** in the *Pfdhps* gene. Mixed infections in the *Pfdhfr* (12) and *Pfdhps* (21) genes were excluded for the haplotype construction. The majority of parasites (84.6%, 143) harboured the triple mutant *Pfdhfr* C**IRN**I haplotype: 89.8% (71) in Klouékanmey and 79.9% (72) in Djougou. The most common *Pfdhps* haplotype was IS**G**KAA (49.6%; 76), followed by the double mutant *Pfdhps* I**AG**KAA (29.3%; 45). The I431**V** mutation was seen in combination with other *Pfdhps* mutations, with three isolates (2.0%) possessing S436**A**, A437**G**, A581**G**, and A613**S**, and one isolate (0.7%) possessing S436**A**, A437**G**, and A613**S**, Table 2.

**Table 2:** Summary of haplotypes observed in *Pfdhfr* and *Pfdhps* genes

	Klouékanmey n (%)	Djougou n (%)	Overall n (%)
*Pfdhfr*			
C**IRN**I	71 (89.8)	72 (79.9)	143 (84.6)
CN**RN**I	1 (1.3)	7 (7.8)	8 (4.7)
CNC**N**C	1 (1.3)	0	1 (0.6)
CNCSI	0	5 (5.6)	5 (3.0)
Mixed-infection	6 (7.6)	6 (6.7)	12 (7.1)
*Pfdhps*			
IS**G**KAA	39 (57.4)	37 (43.5)	76 (49.6)
I**AG**KAA	19 (27.9)	26 (30.6)	45 (29.3)
I**A**AKAA	0	3 (3.5)	3 (2.0)
**VAG**K**GS**	3 (4.4)	0	3 (2.0)
IS**GE**AA	1 (1.5)	1 (1.2)	2 (1.3)
**VAG**KA**S**	0	1 (1.2)	1 (0.7)
I**AG**KA**S**	0	1 (1.2)	1 (0.7)
ISAKAA	0	1 (1.2)	1 (0.7)
Mixed-infection	6 (8.8)	15 (17.6)	21 (13.7)

Bold letters denote mutant alleles

### Combined* Pfdhfr*/*Pfdhps* haplotypes

Combined *Pfdhfr* and *Pfdhps* haplotypes were constructed using 119 samples that were successfully sequenced at each of the codons investigated, Table 3. Mixed infections at any of the codons were excluded. The N51**I**/C59**R**/S108**N**/A437**G** haplotype was found in 106 (89.1%) of the samples. Of these 106, 45 also contained the S436**A** mutation (N51**I**/C59**R**/S108**N**/S436**A**/A437**G**) and one contained the K540**E** mutation (N51**I**/C59**R**/S108**N**/A437**G**/K540**E**). Another two samples contained neither the A437**G** nor the K540**E** mutation but contained the S436**A** mutation (N51**I**/C59**R**/S108**N**/S436**A**). In the samples with the N51**I**/C59**R**/S108**N**/S436**A**/A437**G** haplotype, the A613**S** mutation was found in five samples, three of which also contained the A581**G** mutation.

**Table 3:**
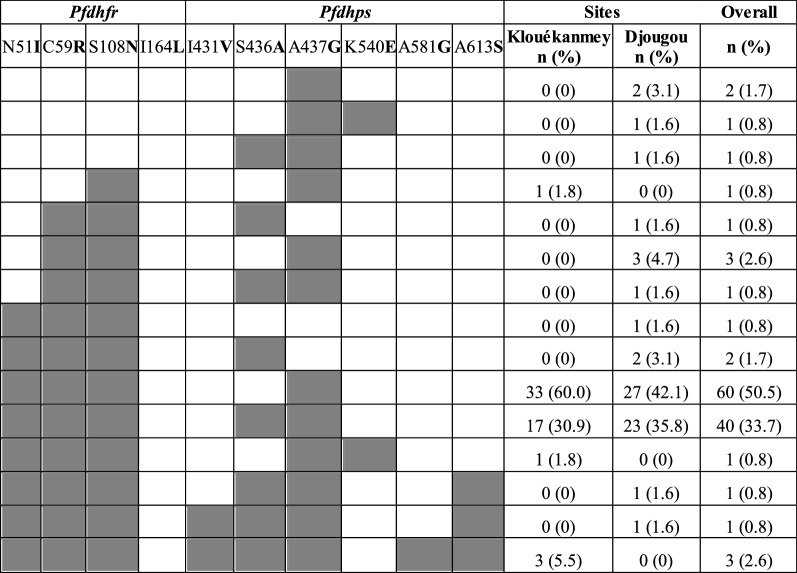
Summary of combined *Pfdhfr* and *Pfdhps* haplotypes. Shaded boxes and bold letters denote mutant alleles. Key haplotypes associated with SP resistance include: the quadruple haplotypes, N51**I**, C59**R**, S108**N** plus S436**A** or A437**G**; the quintuple haplotypes, N51**I**, C59**R**, S108**N **plus A437**G** and K540**E**; or N51**I**, C59**R**, S108**N** plus S436**A** and A437**G**

## Discussion

Results from this study demonstrate that the prevalence of the *Pfdhfr* **IRN** triple mutant is very high, implying these mutants are well established in this region, similar to observations made previously in Benin and in many African countries [16, 18, 39, 40]. In contrast, the prevalence of multiple mutations in the *Pfdhps* gene was low, with the majority of parasites having only a single mutation at codon A437**G** and 29.3% of the parasites with a double mutant (S436**A**/A437**G**), as commonly observed in West Africa [18, 21, 41, 42]. Only a handful of isolates had mutations at codons K540**E** (2.6%), A581**G** (2.6%), and A613**S** (4.6%). A low prevalence or complete absence of these mutations was also observed in other studies in Benin [36, 37]. Several studies have shown the prevalence of these mutations in West Africa is very low compared to East Africa, (reviewed in [16, 18]). This is especially the case for the K540**E** mutation, which has a prevalence greater than 10% in many countries in East Africa but is rarely reported in West Africa [16, 18]. A significant increase in the prevalence of the mutations at codons A581**G** and A613**S** was observed in Nigeria [27, 43], demonstrating the emergence of these mutations. However, their prevalence, even in Benin, is well below the WHO thresholds for consideration of changes in the use of IPTp (> 95% for K540**E** and > 10 % for A581**G**) [44].

Ultimately, the combination of mutations in the *Pfdhfr* and *Pfdhps* genes is one of several factors that determines a parasite’s response to SP. Marked regional differences in the *Pfdhfr* and *Pfdhps* genotypes have been observed across Africa [16, 18]. The quadruple (N51**I**, C59**R**, S108**N** plus A437**G**) mutants are widespread in West Africa, while the quintuple mutants (N51**I**, C59**R**, S108**N** plus A437**G**, 540**E**) and sextuple mutants (addition of *Pfdhps* A581**G** and A613**T**/**S** or *Pfdhfr* I164**L** on the quintuple background) predominate in East Africa [16, 18–20, 38, 45, 46]. In keeping with these observations and with a study conducted in Benin between 2008 and 2010 [37], the majority of isolates in this study were quadruple (N51**I**, C59**R**, S108**N** plus A437**G** ) mutants. The sustained high prevalence of these quadruple mutant parasites is likely due to persistent SP drug pressure from its continual use for IPTp and SMC in Benin. The quintuple (N51**I**, C59**R**, S108**N** plus A437**G** and K540**E)** mutant was observed only in one isolate in Klouékanmey. The minority of isolates in this study with the A581**G** and A613**S** mutations were found in the absence of the K540**E** mutation. These results demonstrate that genotypes conferring a high level of SP resistance have not fully emerged in these study sites, providing support for the continued use of SP for IPTp and SMC in Benin.

A few isolates (2.6 %) in this study possessed the *Pfdhps* I431**V** mutation, which was first described in travelers from Nigeria identified in the UK [47]. A recent study conducted in Nigeria demonstrated an increase in the prevalence of this mutation from 0–6.5 % between 2003 and 2008, and as high as 46 % in 2010 in Enugu, Nigeria [43]. It was also found in pregnant women from other sites in Nigeria such as Epe and Ibeju-Lekki [42]. Furthermore, the I431**V** mutation was seen in isolates from pregnant women in Cameroon and Ghanaian travellers [48, 49], suggesting this mutation is emerging in the region. Interestingly, to date, the I431**V** mutation has not been observed in other parts of Africa. In concordance with previous studies [42, 49], this mutation was observed in combination with other *Pfdhps* mutations (S436**A**/A437**G**/A581**G**/A613**S**), suggesting this mutation may have occurred only in the presence of the other *Pfdhps* mutations. The implications of this combination of *Pfdhps* mutations remain unclear. While some propose these mutations may disrupt the binding of sulfadoxine to the Pfdhps active site [43], additional studies are needed to fully support this notion and to understand the mechanisms involved. Therefore, it is worthwhile to continue monitoring the prevalence of the I431**V** mutation, along with other mutations, in this region.

Limitations of this study include the fact that the samples were obtained from a TES conducted in only two sites in Benin, and therefore the results obtained may not be generalizable to other regions or sites. Moreover, the sample size used was small; additional study sites are recommended for future studies.

## Conclusions

The results from this study indicate that the highly sulfadoxine resistant *Pfdhps* alleles are not widespread in Benin, supporting the current policy of using SP for IPTp and SMC in Benin. However, given the continued use of this drug and limited alternative options, frequent monitoring of SP resistance markers in order to inform IPTp and SMC policies in Benin remains important.

## Data Availability

The full anonymized clinical dataset will be uploaded to Worldwide Antimalarial Resistance Network and WHO repositories upon request and after publication.
